# Molecular, immunological and neurophysiological evaluations for early diagnosis of neural impairment in seropositive leprosy household contacts

**DOI:** 10.1371/journal.pntd.0006494

**Published:** 2018-05-21

**Authors:** Diogo Fernandes dos Santos, Matheus Rocha Mendonça, Douglas Eulálio Antunes, Elaine Fávaro Pípi Sabino, Raquel Campos Pereira, Luiz Ricardo Goulart, Isabela Maria Bernardes Goulart

**Affiliations:** 1 National Reference Center for Sanitary Dermatology and Leprosy, Clinics’ Hospital, School of Medicine, Federal University of Uberlândia (UFU), Uberlândia, MG, Brazil; 2 Postgraduate Program in Health Sciences, School of Medicine, Federal University of Uberlândia (UFU), Uberlândia, MG, Brazil; 3 Institute of Biotechnology, Federal University of Uberlândia (UFU), Uberlândia, MG, Brazil; 4 Department of Medical Microbiology and Immunology, University of California Davis, Davis, CA, United States of America; Kwame Nkrumah University of Science and Technology, GHANA

## Abstract

**Background:**

Household contacts constitute the highest risk group for leprosy development, and despite significant progress in the disease control, early diagnosis remains the primary goals for leprosy management programs.

**Methods:**

We have recruited 175 seropositive and 35 seronegative household contacts from 2014 to 2016, who were subjected to an extensive protocol that included clinical, molecular (peripheral blood qPCR, slit-skin smear qPCR, skin biopsy qPCR) and electroneuromyographic evaluations.

**Results/Principal findings:**

The positivity of peripheral blood qPCR of seropositive contacts was 40.6% (71/175) whereas only 8.6% (3/35) were qPCR positive in seronegative contacts (p = 0.0003). For the slit-skin smear, only 4% (7/175) of seropositive contacts presented positive bacilloscopy, whereas the qPCR detected 47.4% (83/175) positivity in this group compared with only 17.1% (6/35) in seronegative contacts (p = 0.0009). In the ENMG evaluation of contacts, 31.4% (55/175) of seropositives presented some neural impairment, and 13.3% (4/35) in seronegatives (p = 0.0163). The presence of neural thickening conferred a 2.94-fold higher chance of ENMG abnormality (p = 0.0031). Seropositive contacts presented a 4.04-fold higher chance of neural impairment (p = 0.0206). The peripheral blood qPCR positivity presented odds 2.08-fold higher towards neural impairment (OR, 2.08; p = 0.028). Contrarily, the presence of at least one BCG vaccine scar demonstrated 2.44-fold greater protection against neural impairment (OR = 0.41; p = 0.044).

**Conclusions/Significance:**

ELISA anti-PGL-I is the most important test in determining the increased chance of neural impairment in asymptomatic leprosy household contacts. The combination of the two assays (ELISA anti-PGL-I and peripheral blood qPCR) and the presence of BCG scar may identify individuals with higher chances of developing leprosy neuropathy, corroborating with the early diagnosis and treatment.

## Author summary

Despite the apparent progress observed in recent years in leprosy control, early identification of cases remains one of the primary objectives of control programs. In addition, the failure of the current therapeutic scheme on the incidence of leprosy demonstrates that the disease elimination as a public health program promoted by the World Health Organization (WHO) depends on an incisive action to interrupt its transmission chain. The long incubation period of leprosy, its insidious symptoms and signs may difficult its diagnosis. Several studies have recently demonstrated that IgM anti-PGL-I seropositive contacts present higher chances to become ill than seronegative ones. Therefore, our question was: do seropositive contacts at greater risk of becoming sick already present subclinical neural damage? Therefore, our approach was to analyse anti-PGL-I seropositive contacts through electroneuromyography. The development and implementation of more specific and sensitive methods for the detection of *M*. *leprae* and its neural impairment, using immunological, molecular and neurophysiological tools are mandatory to increase the knowledge of leprosy epidemiology, to break its chain of transmission, thereby enabling effective control of this disease. This report demonstrated that seropositive contacts is the population group with higher chances of neural impairment.

## Introduction

Leprosy is a chronic infectious disease caused by *Mycobacterium leprae (M*. *leprae)*, an obligate intracellular parasite with a predilection for infecting peripheral nerves and skin. Leprosy is a current and challenging disease, because it still represents a problem for public health in developing countries such as Brazil, which ranks second worldwide in the number of new cases [1].

The predominance of multibacillary (MB) cases with neural disabilities indicates late diagnosis, reinforcing the ineffective epidemiological control in many countries [2]. In addition, new cases not only with high functional impairment, but also in children, reflect failure of early leprosy detection and indicate ongoing transmission [3,4,5].

Leprosy contacts of MB patients present a risk towards leprosy occurence 5 to 10 times higher than the general population [6,7]. Because of the complex relationships between genetic, immunological and environmental factors, most infected contacts will not develop leprosy, although recent studies have reported that they can be healthy carriers and spread *M*. *leprae* to susceptible individuals [8,9,10].

The investigation of the transmission and infectivity of *M*. *leprae* through molecular and immunological tools has shown that half of the leprosy contacts are healthy carriers, evidenced by the presence of *M*. *leprae* DNA in nasal swabs and, in nasal turbinate biopsies, and/or in the peripheral blood of healthy individuals, while about 18% presented subclinical infection (presence of anti-PGL-I IgM antibodies) with higher risk of illness [10,11,12]. It is important to emphasize that the subclinical neural involvement in this group has not yet been well defined, and its documentation is fundamental. Such elucidation would enable the discussion of chemoprophylaxis and early treatment, as a complementary strategy for leprosy control.

This is a case-control study that aimed to evaluate the clinical and laboratory predictors of subclinical neural impairment in leprosy household contacts.

## Methods

We recruited leprosy household contacts from the National Reference Center of Sanitary Dermatology and Leprosy in Brazil, under the approval of the Ethics Committee of the Federal University of Uberlandia (CAAE: 48293215.7.0000.5152). A written informed consent was obtained from all participants for research participation. Some participants were minors and their parents provided written consent on behalf of them.

At this center, leprosy contacts are followed up for a period of at least 7 years, annually, when they are evaluated by a multidisciplinary team and submitted to dermatoneurological examination and serological (ELISA anti-phenolic glycolipid I Immunoglobulin M; anti-PGL-I IgM) analyses.

In Brazil, epidemiological investigation of contacts consists of an anamnesis addressed to signs and symptoms of leprosy, dermatoneurological examination and vaccination with BCG for contacts without signs and symptoms of leprosy at the time of evaluation, regardless their index case (PB or MB). Application of the BCG vaccine depends on the vaccination history and/or the presence of a vaccine scar, so contacts with no or only one scar should receive a new dose of BCG [7,8].

From 2014 to 2016, 373 new cases of leprosy and 2125 household contacts were reported, totaling an average of 5.7 contacts per patient. A total of 1902 contacts (90.5%, 1902/2125) were examined and 18% (342/1902) were seropositive. In this study, 175 seropositive and 35 seronegative contacts were recruited.

We excluded those who showed clinical evidence of disease (coprevalence cases) and those who presented other etiologies of peripheral neuropathies, such as: chronic alcoholism, diabetes mellitus, thyroid disease, hormonal dysfunctions, malnutrition, hereditary neuropathy, hepatitis B or C, HIV, autoimmune diseases.

### Clinical characterization

Epidemiological and clinical data were recorded. All patients underwent a rigorous dermatoneurological evaluation by expert professionals. Intradermal sensory neuropathy or superficial leprosy neuropathy was defined by the presence of sensory abnormalities in a region not respecting the anatomical distribution of a specific nerve or spinal root, as terminal branches of several nerves may be involved in an affected area, while truncal neuropathy was defined as sensory and/or motor loss respecting the anatomical distribution of a specific nerve or spinal root.

### Laboratory research

Bacilloscopy–analyses of bacillary load of intradermal smears from six sites were performed: the two ear lobes, both elbows and knees, as well as from skin and/or nerve biopsy samples. Sample collection was preceded by topical application of cream containing lidocaine (7%) and tetracaine (7%) at all sites.

ELISA anti PGL-I IgM serology–Enzyme-linked immunosorbent assay (ELISA) was performed on all household contacts. Serum anti-PGL-I IgM antibodies were detected by enzyme-linked immunosorbent assay (ELISA) performed against the purified native PGL-I from the *Mycobacterium leprae* cell wall, according to a methodology previously described elsewhere. The reagent was obtained through BEI Resources, NIAID, NIH: Monoclonal Anti-*Mycobacterium leprae* PGL-I, Clone CS-48 (produced *in vitro*), NR-19370 [13].

DNA Extraction and Real-Time Quantitative Polymerase Chain Reaction (Real-Time PCR)–the DNA extraction from blood (500 μL), slit-skin smear, nerve and skin biopsies were performed. The quantitative real-time PCR (qPCR) assay targeting *M*. *leprae* DNA was performed by targeting the bacillus-specific genomic region (RLEP) in a real-time PCR system (ABI 7300, Applied Biosystems, Foster City, CA, USA) [10,14,15].

### Electroneuromyography

ENMG studies were carried out utilizing the MEB 4200K (NIHON-KODHEN) device. In the sensory conduction study, the median, ulnar, dorsal hand cutaneous, radial, lateral antebrachial cutaneous, median antebrachial cutaneous, sural, fibular superficial, saphenous and medial plantar nerves were examined bilaterally. In the motor conduction study, the median, ulnar, common fibular, and tibial bilaterally nerves were examined, supplemented by techniques for focal impairment identification at compression sites often affected in leprosy neuropathy, such as median nerve at the wrist, ulnar nerve at the elbow, fibular nerve at the fibular head and tibial nerve at the ankle. The parameters used to evaluate each nerve are described separately as a supplementary file.

### Skin biopsy

All of the leprosy contacts selected did not present any skin lesion. For this reason, the biopsy of a small elbow skin fragment (approximately 1 cm) was performed, considering that it is a cold region with possible intradermal impairment, and therefore a site often altered in leprosy neuropathy.

### Nerve biopsy

Nerves that underwent biopsy were selected according to the patient’s clinical condition, and included exclusively sensory nerves that presented sensory changes and/or thickening, and also one of the following electrophysiological changes in the sensory conduction analysis: absence of response on both sides; unilateral absence of response; bilaterally decreased amplitude of the sensory action potential (SAP), considering reference values; and over 50% decrease in the amplitude of the SAP, compared with the contralateral side. During the biopsy, the nerve was isolated and completely transected. All patients signed a specific informed consent form referring to this process. During the procedure, a skin biopsy of the area superjacent to the corresponding territory of the nerve also underwent a biopsy procedure. The biopsied nerve and skin were processed and studied according to routine standard procedures. Formalin-fixed paraffin-embedded were cut longitudinally and transversely at 5-μm thickness and stained with hematoxylin and eosin stain. Additionally, special staining with Masson Trichome was performed to assess fibrosis. Fite-Faraco stain was performed for bacilli identification.

### Statistical analysis

The Shapiro Wilk test was used to test data normality within groups. The Wilcoxon-Mann-Whitney U Test was carried out, and the Binomial Test was applied for the Study of Dichotomous Variables, with significance defined as *p*<0.05. Multiple logistic regression was used to verify the dependence relation between the presence of ENMG abnormality (categorical variable) and the independent variables (ELISA anti-PGL-I IgM, intradermal smear qPCR, skin biopsy qPCR, peripheral blood qPCR and BCG scar). After verifying the dependence between variables, odds ratios (OR) were determined, and the probability of outcomes analyzed. The statistical program used was the software *GraphPad Prism* version *7*.

## Results

Comparisons of all epidemiological characteristics between groups did not show any significant difference (Table 1). The mean anti-PGL-I IgM ELISA index was 2.05 in seropositive contacts, and 0.52 in seronegative contacts (p<0.0001). In the analysis of the peripheral blood qPCR from seropositive contacts, 40.6% (71/175) presented positivity, while only 8.6% (3/35) in seronegative contacts (p = 0.0003). In the intradermal smear analysis, only 4% (7/175) of the seropositive contacts presented positive bacilloscopy, whereas the evaluation by the qPCR in this group showed positivity of 47.4% (83/175) and only 17.1% (6 / 35) in the seronegative contacts (p = 0.0009), all with negative bacilloscopy (Table 1).

**Table 1:** Epidemiological, clinical and laboratory characteristics among the household contacts of leprosy patients.

Parameters	Seropositive household contacts n = 175	Seronegative household contacts n = 35	*p* value
**Age**	30.9±16.2	33.1 ±14.6	0.2297
**Sex**			0.8848
Male	24.0% (42/175)	22.9% (8/35)	
Female	76% (133/175)	77.1% (27/35)	
**Type of contact**			0.4154
Intradomiciliary	76.6% (134/175)	82.9% (29/35)	
Extradomiciliary	23.4% (41/175)	17.1% (6/35)	
**Index case**			1.0
Multibacillary	82.9% (145/175)	82.9% (29/35)	
Paucibacillary	17.1% (30/175)	17.1% (6/35)	
**ELISA index**	2.05 ±0.82	0.52 ±0.18	0.0001
**Peripheral blood qPCR**	40.6% (71/175)	8.6% (3/35)	0.0003
**Slit skin smear qPCR**	47.4% (83/175)	17.1% (6/35)	0.0009
**Bacilloscopy**	4% (7/175)	0.0% (-/35)	-
**Neural thickening**	21.1% (37/175)	8.6% (3/35)	0.0838
**Sensory symptoms (intradermal)**	18.3% (32/175)	14.3% (5/35)	0.5717
**Sensory symptoms (truncal)**	17.1% (30/175)	8.6% (3/35)	0.2079
**Muscular weakness**	2.3% (4/175)	2.8% (1/35)	0.8598
**Abnormal ENMG**	31.4% (55/175)	13.3% (4/35)	0.0163

qPCR = Real Time Quantitative Polymerase Chain Reaction. ELISA = enzyme-linked immunosorbent assay. HHC = household contact. ENMG = Electroneuromyography. BCG = Bacillus Calmette-Guérin.

Regarding the clinical evaluation of seropositive contacts, 18.3% (32/175) presented a pattern of intradermal impairment, compared with 14.3% (5/35) in the seronegative contacts (p = 0.5717), defined as multifocal painful hypoesthesia, especially with a greater involvement in the elbow, knee and earlobe regions, setting a temperature-dependent pattern. Sensorial impairment with a specific territory distribution (truncal pattern) was present in 17.1% (30/175) of seropositive contacts and in 8.6% (3/35) of seronegative contacts (p = 0.2079). The impairment of the deep sensation (vibratory and kinetic postural) and deep osteotendin reflexes was not observed in any case. Only 2.3% (4/175) of seropositive contacts and 2.8% (1/35) of seronegative contacts presented motor manifestation (p = 0.8598). The presence of neural thickening was observed in 21.1% (37/175) of seropositive versus 8.6% (3/35) of seronegative contacts (p = 0.0838). Among contacts with thickening, the ulnar nerve alteration was the most frequently one (72.5%, 29/40). None of the evaluated contacts presented skin lesion.

ENMG evaluation detected some neural impairment in 31.4% (55/175) of seropositive contacts. In seronegative contacts, only 13.3% (4/35) showed changes in this examination (p = 0.0163). (Table 1) Of the 59 contacts with altered ENMG, 81.3% (48/59) were contacts of MB index cases, although this condition did not confer greater chances of alteration in this examination (OR, 0.99; CI95%, 0.45 to 2.15; p = 0.9865).

Only 32.2% (19/59) presented neural thickening in the clinical evaluation. However, the presence of neural thickening conferred a 2.94-fold higher chance of presenting ENMG abnormality (OR = 2.94; CI_95%,_ 1.43 to 6.00; p = 0.0031). The mean number of nerves affected was 1.44 per contact. The nerves most frequently affected are described in Table 2.

**Table 2:** Distribution of peripheral nerves most involved in the electroneuromyographic evaluation of the household contacts of leprosy patients.

Affected Nerves	n	%
Common fibular	33	38.8
Sensory ulnar	23	27.1
Ulnar (Elbow)	11	12.9
Superficial fibular	8	9.5
Sensory median	2	2,3
Superficial radial	2	2,3
Medial antebrachial cutaneous	2	2,3
Sural	1	1,2
Lateral antebrachial cutaneous	1	1,2
Motor median	1	1,2
Deep fibular	1	1,2
**Total nerves**	85	100

In the neurophysiological pattern observed in ENMG, 69.5% (41/59) presented only one altered nerve (mononeuropathy), and 30.5% (18/59) two or more altered nerves (multiple mononeuropathy).

According to clinical data and ENMG results, 50.8% (30/59) of leprosy contacts demonstrated at least one nerve eligible for biopsy, but only 60.0% (18/30) of those were submitted to this process. The most frequent biopsied nerve was the sensory ulnar—dorsal cutaneous of the hand (72.3%; 13/18), followed by superficial fibular (16.7%; 3/18), sural (5.5%; 1/18), and deep fibular (5.5%; 1/18). Only 27.8% (5/18) of the nerves presented some anatomopathological alterations suggestive of leprosy, such as endoneural or epineural infiltrate, presence of fibrosis, perineural thickening or presence of endoneural granuloma. No leprosy contacts presented positive bacilloscopy in the peripheral nerve biopsy. On the other hand, qPCR of nerve biopsies was positive in 61.1% (11/18) of the cases. The qPCR of the suprajacent skin area was positive in 16.7% (3/18) of the nerve biopsies, whereas bacilloscopy was negative in all samples.

In order to further explore the complex interaction among results, a multivariate statistical method was conducted to confirm the dependence relation of variables elucidated above with the chance of occurrence of ENMG abnormalities, demonstrating that ELISA anti-PGL-I positivity confers a 4.04-fold greater chance of neural damage (OR = 4.04; CI_95%_, 1.24 to 13.21; p = 0.020), while peripheral blood qPCR positivity presents a 2.08-fold higher chance (OR = 2.08; CI_95%,_ 1.08 to 4.02; p = 0.028). The presence of at least one BCG vaccine scar demonstrated 2.44-fold greater protection against neural impairment (OR = 0.41; CI_95%,_ 0.18 to 0.98; p = 0.044). There was no dependence relation with the variables intradermal smear qPCR or skin biopsy qPCR (Table 3).

**Table 3:** Analyses of dependence relation between peripheral neural impairment demonstrated by electroneuromyography and the variables ELISA anti-PGL-I, intradermal smear qPCR, skin biopsy qPCR, peripheral blood qPCR and BCG scar, through multiple logistic regression.

Predictor factor	p	ODDS	CI _95%_	Dependence Relation
ELISA anti-PGL-I	0.0206	4.04	1.24–13.21	Yes
Peripheral blood qPCR	0.0288	2.08	1.08–4.02	Yes
Slit skin smear qPCR	0.4186	0.75	0.38–1.49	No
Skin biopsy qPCR	0.9376	0.97	0.46–2.06	No
BCG scar	0.0442	0.41	0.18–0.98	Yes

qPCR = Real Time Quantitative Polymerase Chain Reaction. ELISA = enzyme-linked immunosorbent assay. BCG = Bacillus Calmette-Guérin.

The combination of unfavorable results for the three assays (no BCG scars, seropositivity of anti-PGL-I IgM, and positive qPCR in peripheral blood) indicated the highest probability (62.6%) of neural impairment in contacts. The presence of BCG scars in combination with other disease predictors led to the reduced probability of neural impairment. The group of contacts with favorable results (presence of BCG scars, negative anti-PGL-I and negative qPCR in peripheral blood) was the one with the lowest probability (7.6%) of neural damage (Table 4)

**Table 4:** Probability of peripheral neural impairment demonstrated by electroneuromyography in household contacts of leprosy patients according to predictor factor combinations (ELISA anti-PGL-I, peripheral blood qPCR and BCG scar).

Predictor factor combinations			Probability of neural impairment (%)
ELISA anti-PGL-I	Peripheral blood qPCR	BCG scars	
+	+	-	62.6
+	-	-	44.5
+	+	+	41.2
-	+	-	29.2
+	-	+	25.2
-	-	-	16.5
-	-	+	7.6

qPCR = Real Time Quantitative Polymerase Chain Reaction. ELISA = enzyme-linked immunosorbent assay. BCG = Bacillus Calmette-Guérin.

## Discussion

This is a case-control study in Brazil that measured the chance of occurrence of peripheral neural impairment in asymptomatic leprosy household contacts, through serological, molecular and neurophysiological tests.

The prevalence of abnormalities in the ENMG reinforce the importance of epidemiological surveillance and follow-up of leprosy contacts, allowing early recognition, by a combination of diagnostic tools, of neural impairment in this population.

Previous studies have already documented neural involvement in leprosy contacts, but none has explored how predictors and laboratorial tests are correlated with such pathological occurrence. This is the first study in an endemic country evidencing that subclinical neural impairment may be the first and only clinical manifestation of leprosy, and when appropriately recognized may contribute to early diagnosis and treatment of leprosy, which by definition is primarily neural [16,17,18,19,20].

Some ENMG abnormalities may precede the classic clinical symptoms of leprosy, such as the absence or amplitude reduction of the sensory action potential of some nerves, focal myelinic impairment, which is corroborated by our findings [20–22].

Although asymptomatic, some leprosy contacts already had at least one abnormality detected in the detailed neurologic physical examination, mainly sensory impairment and neural thickening, corroborating the pattern described in the classical forms of leprosy, an asymmetric peripheral neuropathy that is predominantly sensorial. These contacts present a subclinical form in which the ENMG is superior to the thermal, tactile and vibratory sensation evaluation, with capacity for early detection of neural impairment [23,24,25].

Neural thickening, despite being one of the cardinal signs of leprosy and a risk factor for the presence of ENMG abnormalities, as demonstrated in the present study, is a subjective parameter and does not always show agreement with the ENMG, since only one third of the leprosy contacts with ENMG abnormality presented neural thickening [24,25,26].

Leprosy contacts of MB patients did not present higher chances of neural impairment, although this factor is associated with an increase in the disease outcome in several prospective studies [6,9,10,11,12], which only evaluated the natural history of the disease, but without a neurophysiological, serological or molecular intervention for early diagnosis, as shown in our report.

Our results have demonstrated that ELISA anti-PGL-I is the most important test in determining the increased chance of neural impairment in leprosy contacts, corroborating previous studies that also demonstrated its importance as a screening test in the definition of leprosy contacts that present a higher risk of illness. The use of the ELISA anti-PGL-I test is justified due to its high correlation with MB clinical forms, being directly proportional to bacillary load, and also its association with a increased risk of developing leprosy in seropositive contacts [7,10,12,13,27,28,29].

BCG vaccination has been associated with prevention of leprosy in different studies, especially MB forms [7,30]. Based on our results, the presence of one or more BCG scars provided protection against neural damage. Thus, an additional intradermal BCG booster dose should be maintained in leprosy control programs, aiming for protection against leprosy, including neural forms [7].

Concerning the molecular evaluation, studies have shown good prospects regarding the detection of *M*. *leprae* in several samples (blood, skin, swabs, smear) of leprosy patients and contacts by qPCR, which have contributed to the definition of the existence of healthy carriers and subclinical infection [10,11,14,15]. We have shown previously that the positivity of peripheral blood qPCR in contacts was 6.7% with a 5.54-fold risk for disease outcome [10]. Our current results reinforce our previous findings, demonstrating an increased chance of neural involvement in contacts with positive peripheral blood qPCR.

Although the qPCR positivity of intradermic smear and skin biopsy did not determine an increased chance of neural damage, these tools may play a role in diagnostic confirmation, even allowing the initiation of treatment of asymptomatic contacts.

Leprosy household contacts constitute a group of individuals at high risk for disease development, so their participation in the dissemination of *M*. *leprae* to susceptible individuals in endemic communities cannot be neglected [10]. Despite significant progress in controlling leprosy in recent years, early diagnosis remains the primary goal and challenge of leprosy control.

Therefore, with the prospect of eliminating leprosy as a public health problem, the development and implementation of more specific and sensitive methods for the detection of *M*. *leprae* and its neural impairment, using immunological, molecular and neurophysiological tools are mandatory to increase the knowledge of leprosy epidemiology, to break its chain of transmission, thereby enabling effective control of this disease.

**Fig 1:**
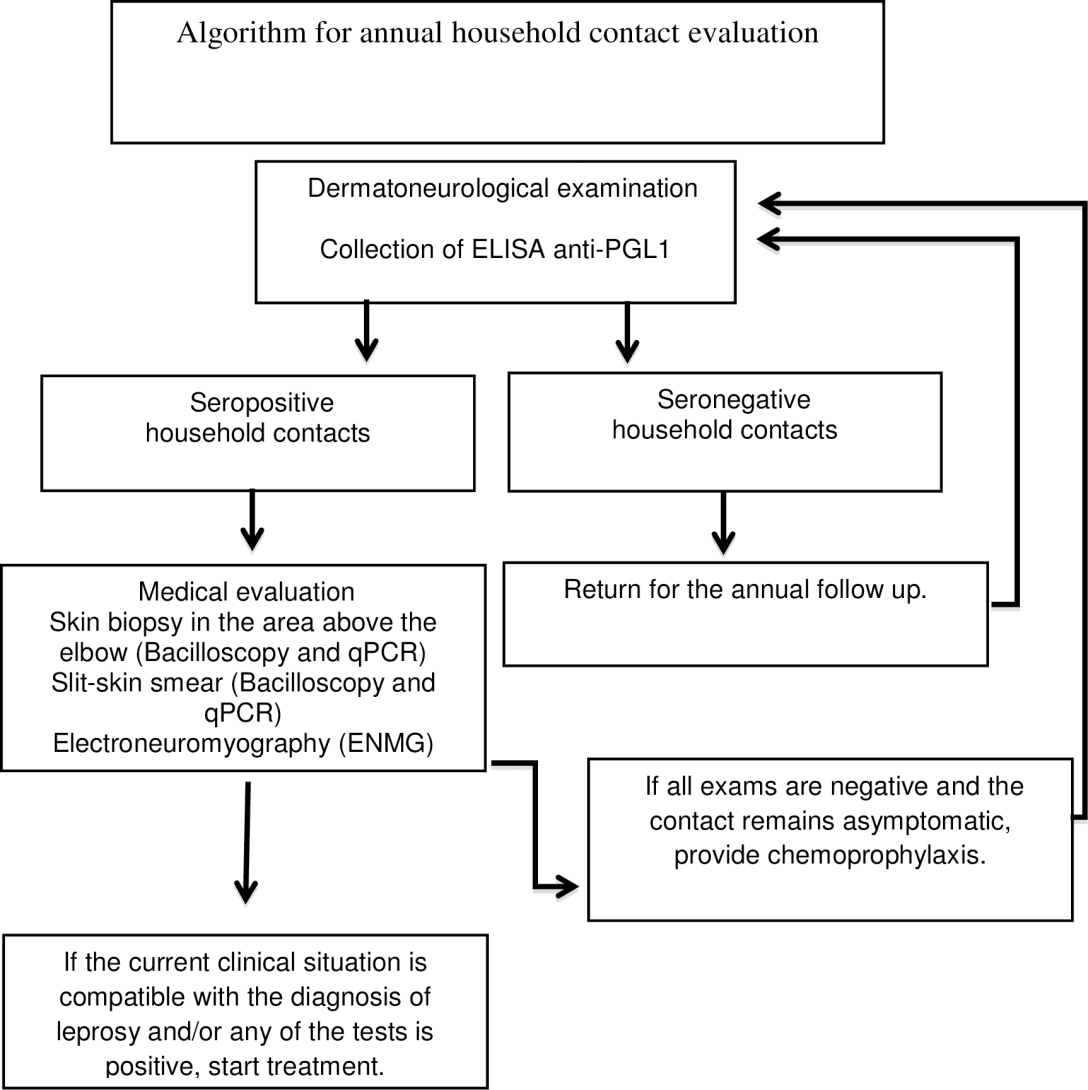
Algorithm proposed for annual household leprosy contacts evaluation. qPCR = Real Time Quantitative Polymerase Chain Reaction. ELISA = enzyme-linked immunosorbent assay. ENMG = electroneuromyography.

Taking into consideration our findings, we propose an algorithm for the follow-up of leprosy household contacts (Fig 1). We suggest annual monitoring through serological (ELISA anti-PGL-I) evaluation for at least 5–7 years, considering the better risk-benefit in relation to neural impairment and development of MB clinical forms [28].

The combination of the three assays in (ELISA anti-PGL-I, peripheral blood qPCR and BCG scars) may identify individuals with higher chances of developing leprosy neuropathy, not only justifying the treatment initiation in those with confirmed diagnosis, but also indicating chemoprophylaxis in contacts with unfavorable predictors.

One of the limitations of the study was that it did not present the follow-up of interventions proposed above, regarding early treatment and chemoprophylaxis, which should be done in future work. In addition, unfortunately, leprosy remains a neglected disease, making it difficult to apply this study to clinical practice in endemic countries.

## Supporting information

S1 File Electroneuromyography. (DOCX)
