# Supplementary material for: Molecular, immunological and neurophysiological evaluations for early diagnosis of neural impairment in seropositive leprosy household contacts
Source: PLoS Negl Trop Dis. 2018 May 21;12(5):e0006494. doi: 10.1371/journal.pntd.0006494 (PMC5983863; doi:10.1371/journal.pntd.0006494)
Supplement: S1 File — (DOCX) [file pntd.0006494.s001.docx]

Electroneuromyography

For motor conduction studies, the gain usually was set at 2 to 5 mV per division. Recording electrodes were placed over the muscle of interest. The active recording electrode (G1) was placed on the center of the muscle belly (over the motor endplate), and the reference electrode (G2) was placed distally, over the tendon to the muscle. The duration of the electrical pulse usually was set to 200 μs, and most nerves required a current sufficient to achieve supramaximal stimulation. For each stimulation site, the latency, amplitude, duration, and area of the compound muscle action potential (CMAP), were measured. A motor conduction velocity were calculated after at least two sites, one distal and one proximal, have been stimulated.

For sensory conduction studies, the gain usually is set at 10 to 20 μV per division. A pair of subdermal needle-recording electrodes (G1 and G2) were placed in line over the nerve at an interelectrode distance of 3 to 4 cm, with the active electrode (G1) placed closest to the stimulator. An electrical pulse of either 100 or 200μs in duration was used, with a current sufficient to achieve supramaximal stimulation. For each stimulation site, the onset latency, peak latency, duration, and amplitude of the sensory nerve action potential (SNAP) were measured. Unlike motor studies, a sensory conduction velocity were calculated with one stimulation alone by taking the measured distance between the stimulator and active recording electrode and dividing by the onset latency.

Both in the sensory conduction study and in the motor conduction study, fixed distances were not established for the stimulation and therefore, the absolute measurements of the distal latencies were not evaluated. Below is a description of the technique used in each of the nerves evaluated:

MEDIAN MOTOR STUDY

Recording Site: Abductor pollicis brevis (APB) muscle (lateral thenar eminence).

Gl placed over the muscle belly.

G2 placed over the first metacarpalphalangeal joint.

Stimulation Sites: Wrist: Middle of the wrist between the tendons to the flexor carpi radialis and palmaris longus. Antecubital fossa: Over the brachial artery pulse.

Amplitude (A) ≥ 4,0 mV; Conduction Velocity (CV) ≥ 50,0 m/s.

MEDIAN SENSORY STUDY

Recording Site: Wrist: G1 placed over the middle of the wrist between the tendons to the flexor carpi radialis and palmaris longus. G2 placed 3-4 cm distally.

Stimulation Sites: Index and middle finger (digit 2 and 3).

A ≥ 10,0 μV, CV ≥ 50,0 m/s.

ULNAR MOTOR STUDY

Recording Site: Abductor digiti minimi (ADM) muscle site (medial hypothenar eminence).

Gl placed over the muscle belly.

G2 placed over the fifth metacarpalphalangeal joint.

Stimulation Sites: Wrist: Medial wrist, adjacent to the flexor carpi ulnaris tendon.

Below elbow: 3-4cm distal to the medial epicondyle.

Above elbow: Over the medial humerus, between the biceps and triceps muscles, at a distance of 10-12 cm from the below-elbow site.

Axilla: In the proximal axilla, medial to the biceps over the axillary pulse.

Amplitude ≥ 6,0 mV; Conduction Velocity ≥ 50,0 m/s.

ULNAR SENSORY STUDY

Recording Site: Wrist: medial wrist, G1 adjacent to the flexor carpi ulnaris tendon. G2 placed 3-4 cm distally.

Stimulation Sites: Little finger (digit 5).

A ≥ 10,0 μV, CV ≥ 50,0 m/s.

DORSAL ULNAR CUTANEOUS SENSORY STUDY

Recording Site: Dorsal hand, G1 over the web space between the little and ling fingers. G2 placed 3-4 cm distally.

Stimulation Sites: Slightly proximal and inferior to the ulnar styloid with the hand pronated.

A ≥ 8,0 μV, CV ≥ 50,0 m/s.

RADIAL SENSORY STUDY

Recording Site: G1 over the superficial radial nerve as it runs over the extensor tendons to the thumb. G2 placed 3-4 cm distally.

Stimulation Sites: Over the distal-mid radius.

A ≥ 15,0 μV, CV ≥ 50,0 m/s.

MEDIAL ANTEBRACHIAL CUTANEOUS SENSORY STUDY

Recording Site: Medial forearm, G1 placed 12 cm distal to the stimulator site, on a line drawn between the stimulation site and the ulnar wrist. G2 placed 3-4 cm distally.

Stimulation Sites: Medial elbow at the midpoint between the biceps tendon and medial epicondyle.

A ≥ 5,0 μV, CV ≥ 50,0 m/s.

LATERAL ANTEBRACHIAL CUTANEOUS SENSORY STUDY

Recording Site: Lateral forearm, G1 placed 12 cm distal to the stimulator site, on a line drawn between the stimulator site and the radial pulse. G2 placed 3-4 cm distally.

Stimulation Sites: Antecubital fossa slightly lateral to the biceps tendon.

A ≥ 10,0 μV, CV ≥ 50,0 m/s.

TIBIAL MOTOR STUDY

Recording Site: Abductor hallucis brevis (AHB) muscle:

G1 placed 1 cm proximal and 1 cm inferior to the navicular prominence.

G2 placed over the metatarsalphalangeal joint of the great toe.

Stimulation Sites: Medial ankle: Above and posterior to the medial malleolus

Popliteal fossa: Mid-posterior knee over the popliteal pulse.

A ≥ 4,0 mV, CV ≥ 40,0 m/s.

PERONEAL MOTOR STUDY

Recording Site: Extensor digitorum brevis (EDB) muscle.

Dorsal lateral foot with G1 placed over the muscle belly.

G2 placed distally over the metatarsalphalangeal joint of the little toe.

Stimulation Sites: Ankle: Anterior ankle, slightly lateral to tibialis anterior tendon.

Below fibular head: Lateral calf, one to two fingerbreadths inferior to fibular head.

Popliteal fossa: Lateral popliteal fossa, adjacent to external hamstring tendons, at a distance of 10-12 cm from the below-fibular head site.

A ≥ 2,0 mV, CV ≥ 40,0 m/s.

SUPERFICIAL PERONEAL SENSORY STUDY

Recording Site: Lateral ankle:

G1 placed between tlle tibialis anterior tendon and lateral malleolus. G2 placed 3-4 cm distally.

Stimulation Sites: Lateral calf

A ≥ 6,0 μV, CV ≥ 40,0 m/s.

SURAL SENSORY STUDY

Recording Site: Posterior ankle: G1 placed posterior to the lateral malleolus. G2 placed 3-4 cm distally.

Stimulation Sites: Posterior-lateral calf.

A ≥ 6,0 μV, CV ≥ 40,0 m/s.

SAPHENOUS SENSORY STUDY

Recording Site: Medial ankle: G1 placed between the medial malleolus and tibialis anterior tendon. G2 placed 3-4 cm distally.

Stimulation Sites: Medial calf.

A ≥ 4,0 μV, CV ≥ 40,0 m/s.

MEDIAL PLANTAR SENSORY STUDY

Recording Site: Medial ankle: G1 placed above and posterior to the medial malleolus. G2 placed 3-4 cm proximally.

Stimulation Sites: Great toe.

A ≥ 2,0 μV, CV ≥ 40,0 m/s.

For the motor conduction analysis, the median, ulnar, common fibular, and tibial bilaterally nerves were examined, supplemented by techniques for focal impairment identification at compression sites often affected in leprosy neuropathy. Segmental stimulation ("inching") of median nerve at the wrist, ulnar nerve at the elbow, fibular nerve at the fibular head and tibial nerve at the ankle were performed. For each l cm increment, latency usually increases 0.2 to 0.3 ms and any abrupt change in latency is suggestive of focal demyelination.

.

Santos DFD, Mendonça MR, Antunes DE, Sabino EFP, Pereira RC, Goulart LR, Goulart IMB. [Revisiting primary neural leprosy: Clinical, serological, molecular, and neurophysiological aspects.](https://www.ncbi.nlm.nih.gov/pubmed/29176796) PLoS Negl Trop Dis. 2017 Nov 27;11(11):e0006086.

Preston, C.; Shapiro, B.E. **Electromyography and neuromuscular disorders**: clinical-electrophysiologic correlations. 2nd ed. 2005.
